# Open questions: completing the parts list and finding the integrating signals

**DOI:** 10.1186/s12915-017-0388-0

**Published:** 2017-06-08

**Authors:** Aurelio A. Teleman, Norbert Perrimon

**Affiliations:** 1 German Cancer Research Center (DKFZ), Im Neuenheimer Feld 580, 69120 Heidelberg, Germany; 2 Department of Genetics, Harvard Medical School, Boston, MA 02115 USA

## Abstract

One of the great revelations of post-genomic biology has been the extent to which essential functions and mechanisms are conserved across vast phylogenetic distances. Because of this, we can look to the fruit fly for answers to pressing open questions on the unknown functions of genes and the mechanisms of their physiological integration.

## To fix a machine, you need to know what the parts do

### Aurelio A. Teleman

Imagine bringing your car to the mechanic for fixing. He opens the hood of the car, reaches in, randomly pulls out a part, and asks with a perplexed look on his face “what does this part do?” Upon hearing this, your hopes of his successfully fixing your car would probably be dashed. This is exactly the situation we are currently facing in the molecular life sciences. We know some molecular or physiological function for roughly half of the genes in the genome - be it the human genome, or the genome of any model organism. For the other half of the genes - or molecular ‘parts’ of the organism, as it were - we are functionally clueless. Although one motivation for doing research in the life sciences is pure curiosity, another motivation is to learn how we can ‘fix’ the human body. With a molecular understanding for the function of only half of all human genes, our chances of successfully fixing the human body in disease is low. Accordingly, we currently have no curative treatment for many types of cancer, for Type 1 or Type 2 diabetes, for neurodegenerative diseases, and so on.

**Fig. 1.**
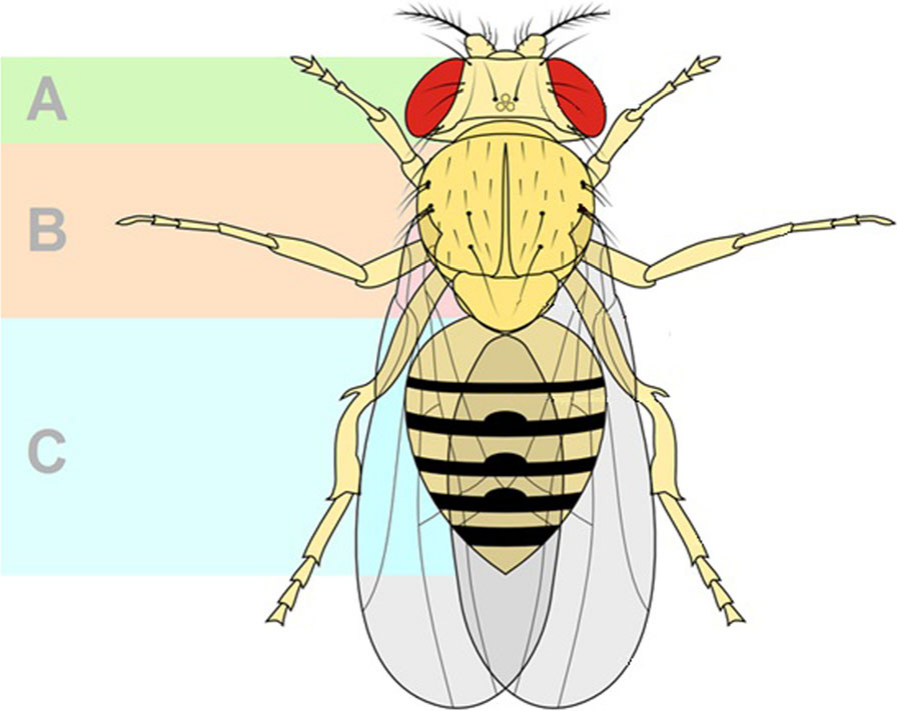
The parts of the fruit fly Drosophila **a**: head; **b**: thorax; **c**: abdomen

Hence an important open question is ‘simply’ to complete the parts list, and elucidate the molecular function of the remaining 50% of the protein-coding genes, and the non-coding RNAs. To date, most of our knowledge regarding the molecular functions of gene products comes from research on model organisms such as yeast, flies, worms and frogs (Fig. 1). For example, genes controlling the eukaryotic cell cycle or autophagy were in large part identified and characterized in yeast [1]. Likewise, research in yeast made fundamental contributions to our molecular understanding of metabolic regulation, transcriptional regulation and protein translation. Research in Drosophila also yielded fundamental discoveries, such as the fact that genes are linked on chromosomes and that X-rays are mutagenic. Many of the components of signalling pathways such as Notch, Wnt or Hippo/YAP were first identified and characterized in Drosophila [2]. Indeed, it appears there are no biological mechanisms that are uniquely human [3], suggesting that model organisms will continue to play an essential role in our ability to efficiently uncover new biology at the molecular level.

Traditionally, gene function has been elucidated in these model organisms via forward genetic approaches, which work by randomly mutagenizing the genome, selecting mutants displaying a phenotype of interest, and then identifying the mutation causing the phenotype. This has worked exquisitely well for phenotypes that can be easily scored. Consequently, many of the genes that yield visually apparent phenotypes when mutated have been identified. The genes that are left uncharacterized are the more ‘difficult’ ones. Indeed, we have noticed that many of the functionally uncharacterized genes that we mutate via targeted approaches nowadays do not yield lethality or visually evident phenotypes. Instead, they yield less obvious phenotypes that are, for instance, quantitative rather than qualitative, metabolic, behavioural, or only apparent under stress conditions. Hence understanding the molecular function of the other 50% of protein-coding genes will be a challenge.

Especially in the current environment where biological research is encouraged to become more ‘translational’, we should keep in mind that to learn human biology, we need to study flies, worms, fish and yeast. Research from these model organisms will give us the biological insights we will need to interpret, for instance, the wealth of human genome sequences that are now at our fingertips.

## Understanding interorgan communication

### Norbert Perrimon

Organ functions are highly specialized. For example, nutrients are taken up through the gut; sensed, processed, stored, and released by the liver and adipose tissues; and utilized by the peripheral organs. Nutrients are used by the skeletal muscle and heart for contraction, by the brain for behavior, by the kidney for water balance and waste disposal, by the gonads for reproduction, and by other tissues for growth. In addition, each organ depends on other organs. For instance, increased physiological demands such as exercise, brain activity, growth, and disease require increased nutrient uptake by the gut and nutrient release (sugars, fats, ketone body) by the liver and adipose tissues. Because organ functions are interdependent, it is important to understand how organs communicate their states to each other, especially as it has implications to diseases and aging.

There is now ample evidence that many organismal functions are involved in mediating various aspects of interorgan communication through secreted hormones [4]. Although a number of hormones have been identified no systematic screen has yet been performed to identify them. Drosophila has emerged in recent years as a prime model to dissect the intricate interactions between organs, especially as tools are available for genome scale interrogation of gene functions. In addition, many physiological regulatory mechanisms are conserved between fly and mammals, and diseases such as obesity and organ wasting/cachexia can be modeled in the fly. Thus, a genetic approach in this model organism should help identify systematically hormones involved in organ communication. Once they have been identified, a key question will be precisely what these hormones sense, whether an acute signal (for example a meal) or a chronic one (for example inflammation), and whether they are they regulated at the transcriptional, translational, or secretion levels. Finally, after elucidating the pathways involved in interorgan communication, the ultimate challenge will be to determine how these pathways integrate with each other in coordinating body functions.
